# The significance of ectopic crypt formation in the differential diagnosis of colorectal polyps

**DOI:** 10.1186/s13000-014-0212-x

**Published:** 2014-11-25

**Authors:** Mi-Jung Kim, Eun-Jung Lee, Sung-Min Chun, Se-Jin Jang, Do Sun Kim, Doo Han Lee, Eui Gon Youk

**Affiliations:** Department of Pathology, Daehang hospital, 481-10 BangBae3-dong, Seoul, Seocho-gu 137-820 Republic of Korea; Department of Surgery, Daehang hospital, Seoul, Republic of Korea; Department of Pathology, University of Ulsan College of Medicine, Asan Medical Center, Poongnap 2-Dong, Seoul, Songpa-Gu 138-736 Republic of Korea

**Keywords:** Traditional serrated adenoma, Conventional adenoma, Colon, Ectopic crypt formation, BRAF, KRAS

## Abstract

**Background:**

Ectopic crypts, defined as abnormally positioned crypts that have lost their orientation toward the muscularis mucosae, have been suggested to be the best defining histologic feature of traditional serrated adenoma (TSA). However, the significance of ectopic crypt formation (ECF) in the distinction between TSA and conventional adenoma (CA) has rarely been studied.

**Methods:**

We designed this study to determine if ECF can be found in CA and its presence is exclusive to TSA. We studied 107 TSAs and 191 CAs including 106 tubular adenomas (TAs), 66 tubulovillous adenomas (TVAs), and 19 villous adenomas (VAs).

**Results:**

ECF was identified in most (79.4%) but not all TSAs. Additionally, ECF was not infrequent in CA (62 of 191, 32.5%), and its presence correlated with the presence of a villous component and larger tumor size (each p <0.001).

**Conclusions:**

Based on its strong association with the presence of a villous component and larger tumor size, ECF appears to be involved in the protuberant growth of colorectal CA. Because ECF can be found in CA, particularly in cases with a villous component, the possibility of CA should be considered before making a diagnosis of TSA when encountering colorectal polyps with ECF.

**Virtual Slides:**

The virtual slide(s) for this article can be found here: http://www.diagnosticpathology.diagnomx.eu/vs/13000_2014_212

## Background

The term traditional serrated adenoma (TSA) refers to a distinct subtype of serrated polyps and was initially proposed for polyps showing a mixture of hyperplastic and adenomatous features [1]. The original definition of TSA referred to a lesion characterized by its overall protuberant exophytic configuration, complex villous growth pattern, and tall columnar cells with abundant eosinophilic cytoplasm and elongated pencillate nuclei [1-3].

Recently, the presence of ectopic crypt formation (ECF), defined as development of abnormally positioned crypts that have lost their orientation toward the muscularis mucosae, was suggested to be the best defining histologic feature to diagnose TSAs based on the report of Torlakovic *et al*. that the presence of ECF was almost exclusive to TSAs among serrated polyps and was observed in all TSA cases [4,5]. However, the diagnostic reliability of ECF in the diagnosis of colorectal polyps, particularly in the distinction of TSAs from other much more common polyps such as conventional adenoma (CA), has rarely been studied [3]. We performed this study to determine whether ECF can be present in CA and whether its presence is a pathognomonic feature in the diagnosis of TSA.

## Methods

### Study sample and histologic evaluation

The present study included 107 TSA samples from 104 patients, all of which were retrieved from the Department of Pathology of Daehang Hospital in Seoul, Korea, between November 2011 and September 2012, as previously described [6]. Of 104 patients, three patients had two TSAs in different location. The lesions were obtained by polypectomy, endoscopic mucosal resection (EMR), endoscopic submucosal dissection (ESD), or transanal excision. Consultation or referral cases from other hospitals were not included in the study. We also studied 191 consecutive CA samples obtained from 188 patients, including 106 tubular adenomas (TAs), 66 tubulovillous adenomas (TVAs), and 19 villous adenomas (VAs), which were completely removed by endoscopic ESD (n = 182) or EMR (n = 9) between January 2011 and September 2012. Among 188 patients with CA, three of them had synchronous CA in different location and underwent ESD for both lesions. None of the CA cases were obtained from patients with TSA. All TSA and CA samples were entirely submitted irrespective of size in order to allow evaluation of histopathologic features without any missing areas.

ECF was defined as abnormal development of crypts with loss of orientation toward the muscularis mucosae [4]. In brief, based on previously published histologic criteria, TSA was characterized by a serrated architecture and at least a focal area containing tall columnar cells with elongated nuclei and abundant eosinophilic cytoplasm [2]. We also investigated features of conventional epithelial dysplasia such as extensive nuclear crowding, nuclear enlargement, loss of nuclear polarity, pseudostratification extending to the upper half of the neoplastic cell, and increased mitoses, according to criteria of previously published studies [7,8]. The size and location of polyps were recorded. The sizes of the polyps were obtained from the pathologist’s measurements. The anatomical sites of polyps were identified and classified as proximal colon (up to the splenic flexure) versus distal colon/rectum.

CA was defined as an adenomatous polyp arranged in a tubular and/or villous architecture. Cytologic dysplasia characterized by the presence of cells with nuclear enlargement, elongation, hyperchromasia, pseudostratification, increased mitoses, and crowding was diffusely identified in the polyp in a top-down manner. The degree of dysplasia was determined according to the WHO classification criteria [3]. CAs were subdivided into TA, TVA, and VA according to the percentage of villous component (<25%, 25-75%, >75%, respectively) [3].

Patients with familial adenomatous polyposis, hereditary nonpolyposis colorectal cancer syndrome, or inflammatory bowel disease were excluded from this study. Patients were assessed for clinicopathologic features, including age and sex, anatomic site and size of the polyp, and the presence and type of synchronous or metachronous sessile serrated adenoma/polyp (SSA/P) upon follow-up by reviewing the patients’ medical records and endoscopy and pathology reports. The slides were stained with hematoxylin and eosin. Tissue collection was approved by the Institutional Review Board at Daehang Hospital.

### DNA extraction

A total of 107 TSA cases containing an adequate amount of tissue in the paraffin blocks were used for molecular studies. In brief, five sections of 10 μm thickness samples were used for genomic DNA extraction. Genomic DNA was extracted using the QIAamp DNA formalin-fixed paraffin-embedded (FFPE) tissue kit (#56404; Qiagen, Hilden, Germany) according to the manufacturer’s instructions. In three CAs with significant numbers of ectopic crypts, we also performed microdissection. Microdissection was performed with great caution using the marked slide as a guide. A razor blade was used to scrape off the cells of interest into a 1.5 ml microcentrifuge tube.

### Detection of mutations for *KRAS* and *BRAF* genes using MassArray technology

As previously described, mutation detection was carried out using an ASAN Panel under the Sequenom MassArray technology platform with the iPLEX-Pro chemistry (Sequenom, San Diego, USA) following the manufacturer’s instructions with minor modifications [6]. In brief, specific assay pools (ASAN Panel) were designed using Assay Designer software in the MassArray Typer package (v4.0). The list of genes and mutations assessed by Sequenom are presented in Table 1. After multiplex PCR with the following program; 95°C for 15 min; 45 cycles of incubations at 95°C for 20 sec, 56°C for 30 sec, 72°C for 30 sec; a final 72°C for 3 min, PCR-amplified DNA was cleansed using a shrimp alkaline phosphatase (SAP) mixture from the iPLEX-Prokit (Cat# 10142-2, Sequenom), and primer was extended by the iPLEX chemistry, desalted using a cation exchange resin (Sequenom) and spotted onto SpectroCHIP II matrix chips using a MassArray nanodispenser. Mass determination was done with the MassArray Analyzer Compact MALDI-TOF mass spectrometer. The MassArray Typer 4.0 software was used for data acquisition and analysis. Genotypes were called after cluster analysis using the default setting of a Gaussian mixture model. Genotype calls were then further reviewed manually to undo any uncertain calls due to clustering artifact. Mutations for a subset of samples and targets were confirmed by Sanger sequencing [6].

**Table 1:** **Detectable mutations using the MassArray method** (**ASAN Panel**)

**Gene**	**Type**	**Exon**	**Mutation**
***KRAS***	Point	Exon 2	G12A
			G12D
			G12V
			G12C
			G12R
			G12S
			G13D
			G13R
			Q61K
			Q61L
			Q61H
***BRAF***	Point	Exon 15	V600E

### Direct DNA sequencing for *KRAS* and *BRAF*

Direct DNA sequencing was performed to verify mutations in *KRAS* and *BRAF*. Polymerase chain reaction (PCR) amplification was performed according to the previously published condition [6,10]. The amplified DNA products were then purified using Montage centrifugal filters (Millipore, Bedford, MA). After purification, direct sequencing was carried out using an ABI PRISM 310 sequence analyzer (Applied Biosystem, Foster city, CA).

### Statistical analysis

Analysis of data was done using SPSS version 21.0 (SPSS Inc., Chicago, IL, USA). For comparing parametric distributions, a Student *t*-test was used, and for frequency distributions, a *χ*^2^ test or Fisher exact test was used. A p value <0.05 was considered statistically significant.

## Results

### Patient demographics and tumor characteristics

A total of 107 TSA cases were obtained from 104 patients comprising 55 males and 49 females with a mean age of 61.2 years. The majority (74.8%) of polyps occurred in the distal colon and rectum, and approximately one-quarter (25.2%) of cases were obtained from the proximal colon. The sizes of the polyps ranged from 0.3 cm to 5.2 cm, with a mean size of 1.3 cm. The size of the lesion differed significantly according to the status of conventional epithelial dysplasia, which was noted in 23 TSA lesions (21.5%); tumors were larger in cases with conventional epithelial dysplasia than in those without (mean; 2.1 cm *vs*. 1.1 cm, p <0.001). Mean age, sex, and tumor location did not differ according to the status of conventional epithelial dysplasia (p = 0.235, p = 1.000, and p = 0.178, respectively).

In total, 191 CAs were obtained from 188 patients: 105 males and 83 females with a mean age of 62.7 years. There was no significant difference between patients with TSA and those with CA with respect to mean age or sex (p = 0.339 and p = 0.229, respectively). Moreover, there were no differences in age or sex among patients with TA, TVA, and VA (p = 0.382 and p = 0.506, respectively). The mean tumor size of CAs was larger than that of TSAs (2.9 cm *vs*. 1.3 cm, p <0.001), most likely because we included CAs obtained by ESD or EMR. Three patients with CA had a prior history of right-sided SSA/Ps, and 10 other patients with CAs had synchronous or metachronous SSA/P. All 13 cases of SSA/P were histologically proven. The clinicopathologic features of the patients with TSA and CA are summarized in Table 2.

**Table 2:** **Patient demographics and tumor characteristics**

		**CA group**			
**Parameters**	**TSA group (n = 107)** ^**a**^	**TA(n = 106)** ^**b**^	**TVA (n = 66)** ^**b**^	**VA (n = 19)**	**p**
**Mean age (y)**	61.2	63.3	62.6	59.7	0.345
(range)	(30-81)	(31-85)	(37-80)	(34-79)	
**Gender**					0.634
Woman	49	42	31	10	
Man	55	62	34	9	
**Tumor location**					<0.001
Proximal	27 (25.2%)	62 (58.5%)	30 (45.5%)	2 (10.5%)	
Distal	80 (74.8%)	44 (41.5%)	36 (54.5%)	17 (89.5%)	
**Mean tumor size (cm)**	1.3	2.7	3.2	3.6	<0.001
**Procedure**					NA
Polypectomy	37 (34.6%)	0	0	0	
EMR	57 (53.3%)	5 (4.7%)	4 (6.1%)	0	
ESD	12 (11.2%)	101 (95.3%)	62 (93.9%)	19 (100%)	
Transanal excision	1 (0.9%)	0	0	0	

TSA, traditional serrated adenoma; CA, conventional adenoma; TA, tubular adenoma; TVA, tubulovillous adenoma; VA, villous adenoma; NA, not applicable; EMR, endoscopic mucosal resection; ESD, endoscopic submucosal dissection.

^a^107 TSAs from 104 patients.

^b^191 CAs from 188 patients.

### Histologic findings

**Figure 1:**
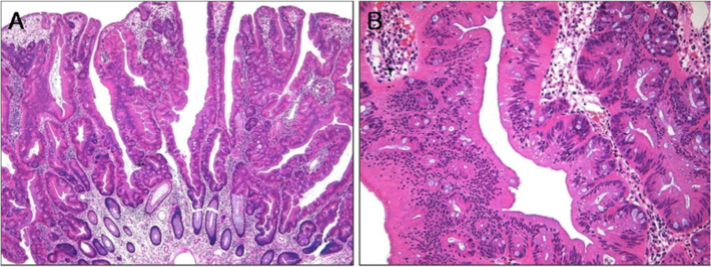
**Histologic findings of traditional serrated adenoma (TSA). (A)**, TSA showing a protuberant growth pattern and serrated architecture (hematoxylin and eosin; original magnification × 40). **(B)**, TSA composed of tall columnar cells with diffuse serration and abundant eosinophilic cytoplasm. Note the abundant ectopic crypts (hematoxylin and eosin; original magnification × 200).

All of the cases of TSA included in this study exhibited a serrated architecture in at least 20% of the tumor area and at least one focal area of tall columnar cells with abundant eosinophilic cytoplasm (Figure 1A). The low-power images of TSAs were somewhat variable depending on the growth pattern, the extent of peculiar tall columnar cells with eosinophilic cytoplasm, the status of precursor lesions, and the amount of stromal component. ECF was identified in most (79.4%) but not all cases of TSA (Figure 1B). The frequency of ECF varied depending on the case or even the area within the same case. ECF was numerous in 15.0% of cases, whereas some cases (5.7%) displayed minimal ECF.

**Figure 2:**
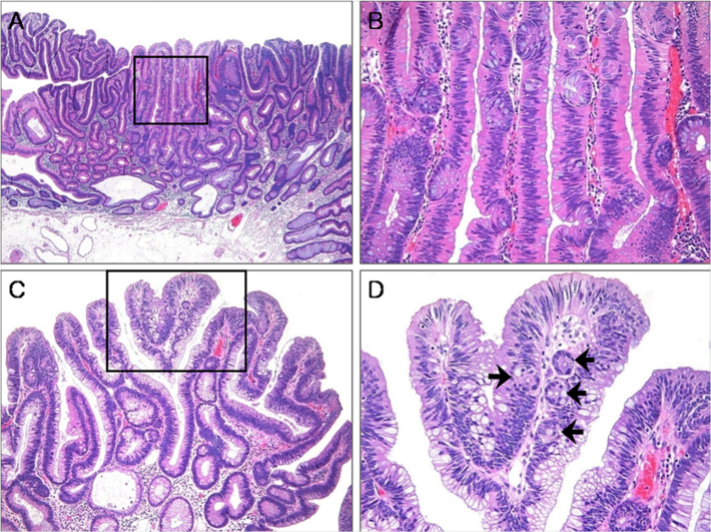
**Conventional adenoma (CA) with a focal villous architecture. (A)**, Tubular adenoma (TA) composed of columnar cells with diffuse conventional dysplasia and focal epithelial serration (hematoxylin and eosin; original magnification × 40). **(B)**, Higher magnification of the box in panel A, showing dysplastic cells with cytoplasmic eosinophilia and ectopic crypts (hematoxylin and eosin; original magnification × 200). **(C)**, Tubulovillous adenoma (TVA) with a villous architecture and columnar cells with diffuse conventional dysplasia (hematoxylin and eosin; original magnification × 100). **(D)**, Higher magnification of the box in panel C, displaying ectopic crypts (arrows) (hematoxylin and eosin; original magnification × 200).

**Figure 3:**
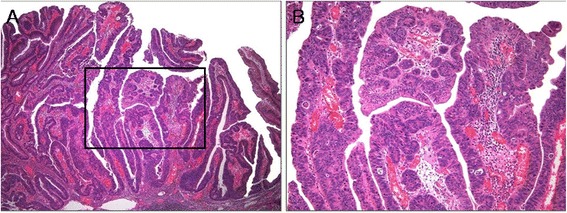
**Tubulovillous adenoma (TVA) with high grade dysplasia. (A)**, TVA with a villous architecture and columnar cells showing diffuse high grade dysplasia (hematoxylin and eosin; original magnification × 40). **(B)**, Box in panel A, displaying high grade dysplasia and ectopic crypts (hematoxylin and eosin; original magnification × 100).

**Figure 4:**
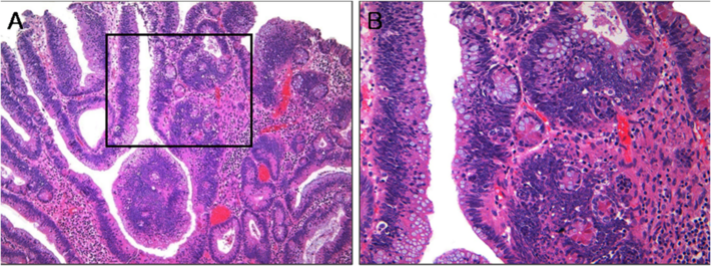
**Tubulovillous adenoma (TVA) with ectopic crypts and Paneth cell metaplasia. (A)**, TVA with frequent ectopic crypts (hematoxylin and eosin; original magnification × 100). **(B)**, Higher magnification of the box in panel A, displaying ectopic crypts showing Paneth cell metaplasia (hematoxylin and eosin; original magnification × 200).

ECF was present in 62 (32.5%) cases of CA (Figures 2 and 3). The frequency of ECF in TAs, TVAs, and VAs was 22.6% (24 cases), 37.9% (25 cases), and 68.4% (13 cases), respectively (p <0.001) (Table 3). ECF was sparse in all but three cases of CA. Most CAs had amphophilic or basophilic cytoplasm, although a minority (5.2%) showed abundant eosinophilic cytoplasm in focal areas that was significantly associated with the presence of ECF (p <0.001) (Figure 2A and B). The presence of ECF significantly correlated with the presence of villosity and large tumor size (each p <0.001; Figure 2C and D). ECF was also noted in CA with high-grade dysplasia (HGD) as well as that with low-grade dysplasia (LGD) (Figure 3A and B). Serration of crypts was noted in 20 (10.5%) cases of CA; however, epithelial serration accounted for less than 5% of the polyp area in all but one CA, in which serration of crypts occupied approximately 20% of the area. In that case, the epithelial serration was concentrated in the middle and lower portions of crypts associated with dilatation. Three CA cases showed Paneth cell metaplasia in areas with ECF (Figure 4A and B). In 16 CA cases, ECF, focal serration, and cytoplasmic eosinophilia occurred in the same polyp. The presence of ECF was significantly associated with focal serration of crypts and cytoplasmic eosinophilia in CA (each p <0.001).

**Table 3:** **Comparison of histologic features according to the subtype of CAs**

**Parameters**	**TA (106), n(%)**	**TVA (66), n(%)**	**VA (19), n(%)**	**p**
ECF (%)	24 (22.6%)	25 (37.9%)	13 (68.4%)	< 0.001
Focal serration (%)	7 (6.6%)	7 (10.6%)	6 (31.6%)	0.005
Focal cytoplasmic eosinophilia (%)	1 (0.9%)	4 (6.1%)	5 (26.3%)	< 0.001

CA, conventional adenoma; ECF, ectopic crypt formation; TA, tubular adenoma; TVA, tubulovillous adenoma; VA, villous adenoma.

### Mutation results for *BRAF* and *KRAS* genes

**Figure 5:**
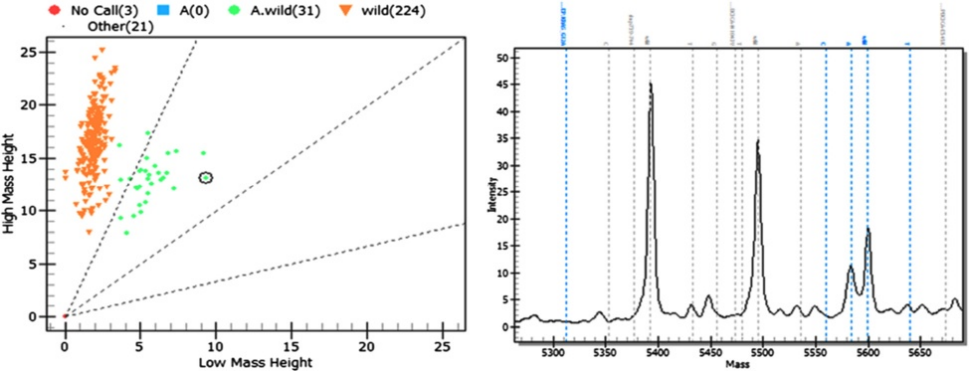
**Mutational profiling using the Sequenom MassArray platform.** Sequenom MassArray identifying the *KRAS* 12G > A mutation.

*BRAF* mutation was observed in 59 (55.1%) TSA lesions, and *KRAS* mutations were identified in 36 (33.6%) cases. Mutations of *BRAF* and *KRAS* were mutually exclusive. Twelve (11.2%) TSA lesions showed neither *BRAF* 600E nor *KRAS* codon 12, 13, or 61 mutations. Among three cases of CA showing significant numbers of ectopic crypts, two showed *KRAS* mutation, while the other displayed neither *BRAF* nor *KRAS* mutation (Figure 5).

## Discussion

TSA is a distinct subtype of serrated neoplasm that is usually characterized by a protuberant growth pattern with an overall complex and often villous growth pattern, epithelial serration, and tall columnar cells with abundant eosinophilic cytoplasm and elongated pencillate nuclei [1-3]. However, the diagnosis of TSA is sometimes challenging because of overlap with other polyps in terms of both gross and histologic features. More specifically, the gross morphology does not distinguish TSAs from other polyps because TSAs vary from flat elevated and sessile to pedunculated forms [9]. A pedunculated configuration, which is known to be frequent in TSAs, is also found in other polyps, in particular the so-called ‘pedunculated serrated polyp with SSA/P features’ and CA [10]. The former lesion is a newly described serrated polyp that has clinicopathologic features of SSA/P except for the pedunculated gross configuration. The complex villous growth pattern can also be identified in CA. Serration of crypts can be observed in various conditions such as inflammatory bowel disease, juvenile polyps, inflammatory polyps, and CAs, as well as other kinds of serrated polyps [11-13]. In addition, the characteristic cytological features such as abundant eosinophilic cytoplasm and pencillate nuclei can be found in other serrated polyps such as SSA/P or sessile serrated adenoma with dysplasia (SSA/D), as well as in CA [14]. In summary, none of these histopathologic features alone is considered specific for TSA. Therefore, in this study, we defined TSA as a lesion combining a serrated architecture and at least one focal area containing tall columnar cells with elongated nuclei and abundant eosinophilic cytoplasm [2].

**Figure 6:**
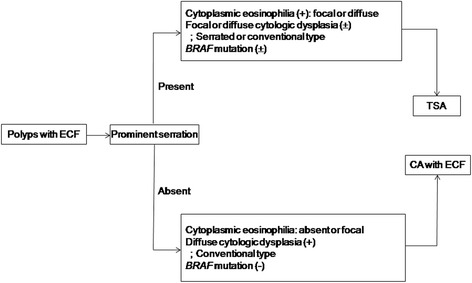
**Schematic representation of the diagnosis of polyps showing ectopic crypt formation (ECF).**

Recently, the presence of ECF has been suggested to be the best defining histologic feature to diagnose TSA [4,5]. However, the presence of ectopic crypts, which was initially suggested to be exclusive to TSA, was reported to be present in other serrated polyps such as HP or SSA/P [14]. Similar to the result by Ensari *et al*. [14], ECF was noted in approximately 80% of TSA cases in the present study. In addition, a considerable fraction of CA showed ECF, albeit most cases were sparse. Although TSA showed a higher frequency of ECF than CA (p <0.001), the frequency of ECF in CA (32.5%) was higher than we expected, particularly in cases with a villous component (p <0.001). The actual rate of ECF, particularly in polypectomy specimens, might be lower than that of the present study because we included large CAs obtained by ESD or EMR. At any rate, ECF itself does not seem to be a *sine qua non* for the diagnosis of TSA because ECF can be found in CA, although its identification might be very helpful in the diagnosis of TSA. Therefore, if one encounters colorectal polyps with ECF, the possibility of CA should always be considered. In such cases, histologic evaluation for the presence and/or extent of crypt serration, extent of ECF, and cytoplasmic characteristics are necessary to distinguish TSA from CA with ECF (Figure 6). Absence of extensive ECF, prominent serration, serrated dysplasia, and/or diffuse cytologic eosinophilia are histologic features favoring a diagnosis of CA over TSA. According to the results of this study, the extent of serration did not exceed 20% of crypts in CA.

In addition to the histopathologic evaluation, the results of molecular studies for *BRAF* mutation are also important in the differentiation of CA from TSA because *BRAF* mutation is rare in CA (<1%) but common in TSA [15-17]. Mutation study for *KRAS* seems to be less useful than *BRAF* testing in the distinction between CA and TSA, although the frequency of *KRAS* mutations is lower in TSA than in CA [17]. In the present study, we performed mutation study for *KRAS* and *BRAF* in order to investigate whether *BRAF* mutation was present in CA with frequent ECF. As expected, none of these cases exhibited a *BRAF* V600E mutation. Instead, two of three CAs with frequent ECF showed *KRAS* mutations. Mutation study of the *APC* gene might be also helpful in the distinction between CA and TSA because, in contrast to CA, *APC* gene mutation is infrequent in TSA [18]. However, we could not perform molecular study of the *APC* gene in this study.

The presence of ECF, serration of crypts, and cytoplasmic eosinophilia in some CAs suggest the possibility that such polyps share a series of molecular alterations with TSAs that are responsible for the histological differentiation toward serrated neoplasia. Accordingly, Pai *et al*. [19] reported that a subset of CA shows atypical features such as cytoplasmic eosinophilia with or without focal serrations, usually in patients predisposed to developing SSA/P. On molecular analysis, these atypical CAs showed a higher frequency of CpG island methylation than did typical CA (35.7% *vs*. 4.2%). We did not find any significant difference in the rates of ECF, cytoplasmic eosinophilia, or serration of crypts between CA that developed SSA/P and CA that did not (p = 0.228, p = 0.515, and p = 1.000, respectively), although this might be because of the small number of patients that developed SSA/P among the patients with CA.

We believe that CA with ECF is not uncommon, although there is some controversy over whether such cases represent TSA with diffuse conventional dysplasia [5]. Indeed, most CAs in this study were otherwise typical, except for three cases that showed easily recognizable ECF, none of which showed prominent serration of crypts (more than 20%), serrated dysplasia, diffuse cytoplasmic eosinophilia, or *BRAF* mutation. According to a previous study, ECF is considered one of the mechanisms that increase crypt numbers as a result of complex biologic processes involving a sequential proliferation-differentiation process that depends on the time point and epithelial-mesenchymal interaction [20]. It is presumed that inhibition of bone morphogenic protein (BMP) signaling results in the formation of ectopic crypt units perpendicular to the crypt-villus axis [21]. The concentration of Ki-67^+^ cells in ectopic crypts supports our contention that ectopic crypts play an important role in the protuberant growth of polyps [4]. Accordingly, the presence of ECF significantly correlated with large tumor size in this study.

## Conclusions

In conclusion, ECF appears to be associated with villous or protuberant growth of colorectal adenoma. Although identification of ECF seems to be very helpful in the diagnosis of TSA, ECF is also found in CA, particularly in cases with a villous component. Therefore, the possibility of CA should be considered before making a diagnosis of TSA in cases of colorectal polyp with ECF.

## Abbreviations

BMP: Bone morphogenic protein
CA: Conventional adenoma
ECF: Ectopic crypt formation
EMR: Endoscopic mucosal resection
ESD: Endoscopic submucosal dissection
FFPE: Formalin-fixed paraffin-embedded
HGD: High grade dysplasia
LGD: Low grade dysplasia
PCR: Polymerase chain reaction
SSA/P: Sessile serrated adenoma/polyp
TA: Tubular adenoma
TSA: Traditional serrated adenoma
TVA: Tubulovillous adenoma
VA: Villous adenoma
